# Design of Over-1000 nm Near-Infrared Fluorescent Polymeric Micellar Nanoparticles by Matching the Solubility Parameter of the Core Polymer and Dye

**DOI:** 10.1021/acsnanoscienceau.1c00010

**Published:** 2021-10-04

**Authors:** Yuichi Ueya, Masakazu Umezawa, Yuka Kobayashi, Hisanori Kobayashi, Kotoe Ichihashi, Takashi Matsuda, Eiji Takamoto, Masao Kamimura, Kohei Soga

**Affiliations:** † Tsukuba Research Laboratories, JSR Corporation, 25 Miyukigaoka, Tsukuba, Ibaraki 305-0841, Japan; ‡ Department of Materials Science and Technology, Faculty of Advanced Engineering, Tokyo University of Science, 6-3-1 Niijuku, Katsushika, Tokyo 125-8585, Japan

**Keywords:** Bioimaging, Second infrared window, Hansen solubility parameter, Polymeric micellar nanoparticles, Fluorescent dye

## Abstract

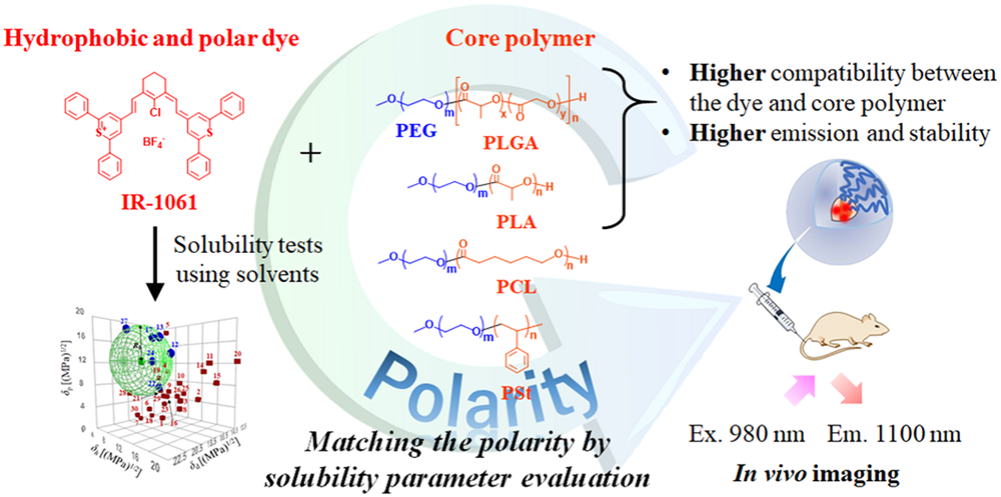

Polymeric micellar nanoparticles (PNPs) encapsulating over-thousand-nanometer (OTN) near-infrared (NIR) fluorescent dye molecules in block polymers having hydrophobic and hydrophilic chains are promising agents for the dynamic imaging of deep tissue. To achieve OTN-NIR fluorescent PNPs (OTN-PNPs) having high brightness, it is crucial to increase the affinity between the core polymer and dye molecules by matching their polarities; thus, criteria and methods to evaluate the affinity are required. In this study, we used the Hansen solubility parameter (HSP), including the polarity term, to evaluate the affinity between the two substances. HSP values of the OTN-NIR fluorescent dye IR-1061 and four core polymers, poly(lactic-*co*-glycolic acid) (PLGA), poly(lactic acid) (PLA), poly(ε-caprolactone) (PCL), and polystyrene (PSt), were calculated using the Hansen solubility sphere method and molecular group contribution method, respectively. The relative energy density between IR-1061 and each core polymer calculated using their HSP values revealed that the affinities of PLGA and PLA for IR-1061 are higher than those of PCL and PSt. Therefore, OTN-PNPs composed of PLGA, PLA, and PCL core polymers were prepared and compared. The OTN-PNPs having PLGA and PLA cores could be loaded with larger amounts of IR-1061, had higher photoluminescence intensities, and showed higher stability in phosphate buffered saline than those having PCL cores. Moreover, the OTN-PNPs having PLGA or PLA cores were used for the dynamic imaging of live mice. Thus, matching the solubility parameters of the core polymer and dye molecule is a useful approach for designing high-performance OTN-NIR fluorescent probes.

## Introduction

Dynamic live imaging in the over-thousand-nanometer (OTN) near-infrared (NIR) optical window, the so-called second biological window (NIR-II),^1^ allows significantly higher spatial resolution with deeper imaging depth^2−4^ and lower autofluorescence^5^ than imaging in the traditional visible (400–700 nm) and first NIR window (NIR-I, 700–900 nm). Among the various OTN-NIR fluorescent imaging probes, including rare-earth-doped ceramics,^6−8^ single-walled carbon nanotubes,^9−12^ and quantum dots,^13−15^ organic-dye-based fluorophores exhibit great potential for biomedical research and clinical use because of their high biocompatibility, designable structures, and tunable optical properties.^16−18^

**Figure 1:**
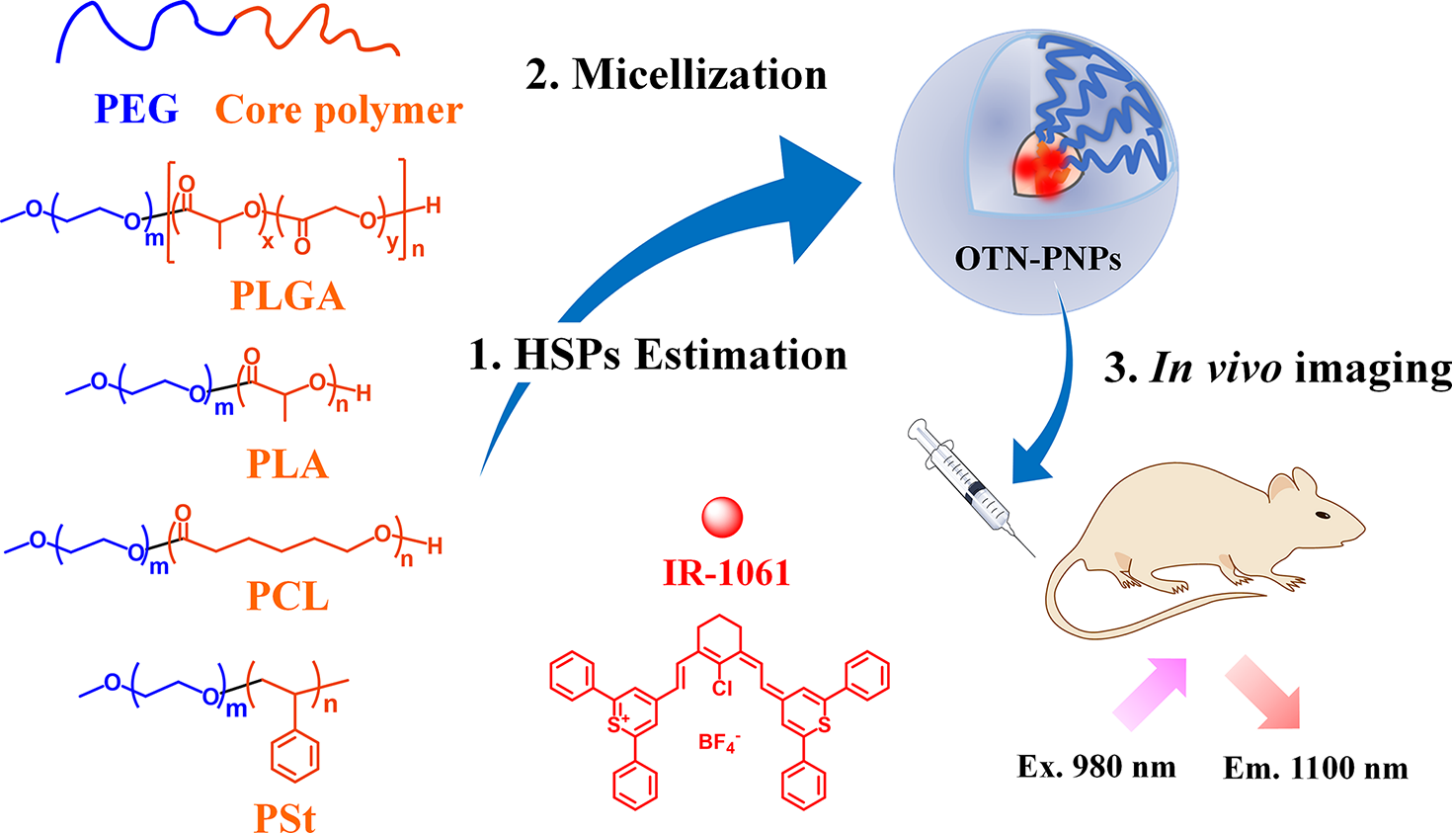
Molecular structures of the compounds studied and schematic of the OTN-PNPs (Ex. = excitation, Em. = emission).

Polymethine molecules such as IR-26, IR-1048, IR-1051, and IR-1061 are commercially available OTN-NIR fluorophores. They need to be conjugated or incorporated into biocompatible polymers for use in *in vivo* imaging because they are insoluble in water. While polymer formulations of various dyes such as phthalocyanines^35^ and heptamethines^36,37^ have been investigated, the encapsulation of IR-1061 in biocompatible polymer nanoparticles (PNPs) for use in *in vivo* imaging has also been reported.^19,20^ Specifically, IR-1061 (see Figure 1) is a polar and hydrophobic molecule that undergoes aggregation and fluorescence quenching in environments with mismatched polarities. For example, recently, we reported highly emissive OTN-NIR polystyrene (PSt)-based nanoparticles having core polymers with tuned polarities, which was achieved by changing the monomer ratio (styrene to acrylic acid), to increase the affinity for IR-1061.^21^ However, the quantitative evaluation of the affinity of dyes for the core polymers remains challenging, and this has limited the rational and efficient design of OTN-NIR fluorescent agents.

One possible tool for the numerical estimation of the affinity between two substances is the solubility parameter. There are two common solubility parameters: the Hildebrand solubility parameter and the Hansen solubility parameter (HSP). The Hildebrand solubility parameter condenses all structural information into one representative value, whereas the HSP is composed of three factors: dispersion forces (δ_d_), intermolecular dipole interactions (δ_p_) and hydrogen-bonding interactions (δ_h_).^22^ Therefore, in this study, we chose the HSP to enable the better approximation of polar interactions and hydrogen bonding between the two substances, that is, the organic polymer and fluorescent dye.^23−25^

We first investigated the HSPs of the polar dye molecule IR-1061 and the hydrophobic parts of the amphiphilic polymers that interact with IR-1061 in the OTN-PNPs. We selected four commercially available amphiphilic polymers: poly(ethylene glycol) (PEG)-*block*-poly(lactic-*co*-glycolic acid) (PLGA) (PEG-*b*-PLGA), PEG-*block*-poly(lactic acid) (PLA) (PEG-*b*-PLA), PEG-*block*-poly(ε-caprolactone) (PCL) (PEG-*b*-PCL), and PEG-*block*-PSt (PEG-*b*-PSt) (see Figure 1 for the corresponding structures). To design OTN-PNPs with a high affinity between IR-1061 and the core polymer, it is necessary to evaluate the solubility of solid IR-1061 and each core polymer quantitatively. Thus, we attempted to determine whether solid IR-1061 and the core polymer could dissolve together by examining the relative energy difference (RED) calculated from the corresponding HSPs. To validate the affinities predicted by HSP evaluation, we confirmed the IR-1061 content encapsulated in the OTN-PNPs, as well as the brightness and stability of the OTN-PNPs. Furthermore, *in vivo* imaging of mice was performed using OTN-PNPs prepared from PEG-*b*-PLGA and PEG-*b*-PLA, which were found to have a high affinity for IR-1061 in our HSP evaluation.

## Experimental Section

### Materials

IR-1061 and PEG-*b*-PLGA (*M*_n_: 2000–4500, lactide/glycolide = 65:35) were purchased from Sigma-Aldrich (St. Louis, MO, USA). PEG-*b*-PLA (*M*_n_: 2000–5000) and PEG-*b*-PCL (*M*_n_: 2000–5000) were purchased from NOF Corporation (Tokyo, Japan). Acetonitrile (ACN) and Dulbecco’s phosphate buffered saline (PBS) were purchased from Fujifilm Wako Pure Chemical Corporation (Osaka, Japan). Bovine serum albumin was purchased from Hoffmann-La Roche (Basel, Switzerland). All reagents were used without further purification.

### HSP Determination of IR-1061 and Core Polymers

The HSP of IR-1061 was determined using Hansen Solubility Parameters in Practice (HSPiP)^26^ based on the Hansen solubility sphere method.^27^ In the Hansen solubility sphere method, the HSP is calculated by testing the solubility of the unknown material in various organic solvents. This method can be used regardless of the state of the sample; therefore, it is suitable for determining the HSP of ionic compounds such as IR-1061. Therefore, we evaluated the affinity of IR-1061 for typical organic solvents whose HSPs are already known.^26^

One milliliter of each of the 30 organic solvents listed in Table 1 was added to 2 mg of IR-1061. After sonication for 10 min in a bath sonicator (UT-206, SHARP, Japan), each tube was centrifuged at 21 500 *g* for 10 min (CF16 RN, HITACHI, Japan). If a precipitate of IR-1061 was observed, the solvent was judged to have a low affinity for IR-1061 with a score of 0, whereas when IR-1061 was completely dissolved, the solvent was judged to have a high affinity with a score of 1. From the solubility data, each organic solvent was plotted in the HSP space, and a fitting procedure was used to determine the HSP sphere of IR-1061. The center coordinates and radius of the HSP sphere were obtained by using software (HSPiP, 4th ed., 4.0.07) from the results of solubility scores of IR-1061 in each solvent. In addition, the radius of the HSP sphere is the interaction radius (*R*_0_) of IR-1061, which is a tolerance indicator for the interaction of IR-1061 with other materials.^28^

**Table 1:** Solubility Scores of IR-1061 in 30 Organic Solvents and HSPs of Each Organic Solvent

	solvents	δ_d_ (MPa)^1/2^	δ_p_ (MPa)^1/2^	δ_h_ (MPa)^1/2^	solubility score^a^
1	1, 4-dioxane^b^	19.0	1.8	7.4	0
2	1-butanol^c^	16.0	5.7	15.8	0
3	2-phenoxy ethanol^d^	17.8	5.7	14.3	0
4	acetone^c^	15.5	10.4	7.0	0
5	acetonitrile^c^	15.3	18.0	6.1	0
6	chloroform^c^	17.8	3.1	5.7	0
7	cyclohexane^c^	16.8	0.0	0.2	0
8	cyclohexanol^c^	17.4	4.1	13.5	0
9	dibasic ester (DBE)^d^	16.2	4.7	8.4	0
10	diacetone alcohol^d^	15.8	8.2	10.8	0
11	diethylene glycol^b^	16.6	12.0	20.7	0
12	*N*,*N*-dimethylformamide^c^	17.4	13.7	11.3	1
13	dimethyl sulfoxide^c^	18.4	16.4	10.2	1
14	dipropylene glycol^b^	16.5	10.6	17.7	0
15	ethanol^c^	15.8	8.8	19.4	0
16	ethyl acetate^c^	15.8	5.3	7.2	0
17	γ-butyrolactone^b^	19.0	16.6	7.4	1
18	hexane^c^	14.9	0.0	0.0	0
19	methyl ethyl ketone^d^	16.0	9.0	5.1	0
20	methanol^c^	15.1	12.3	22.3	0
21	methyl isobutyl ketone^b^	15.3	6.1	4.1	0
22	methylene dichloride^d^	18.2	6.3	6.1	1
23	*n*-butyl acetate^b^	15.8	3.7	6.3	0
24	*N*-methyl-2-pyrrolidone^d^	18.0	12.3	7.2	1
25	propylene glycol methyl ether^b^	15.6	6.3	11.6	0
26	methoxy propyl acetate^b^	15.6	5.6	9.8	0
27	propylene carbonate^b^	20.0	18.0	4.1	1
28	tetrachloroethylene^c^	18.0	5.0	0.0	0
29	tetrahydrofuran^c^	16.8	5.7	8.0	0
30	toluene^b^	18.0	1.4	2.0	0

a Score = 0: low-affinity solvents for IR-1061; score = 1: high-affinity solvents for IR-1061. The HSPs of each solvent are cited from ref (26). The manufacturers from which we purchased each solvent are shown in the “solvent” column as follows.

b TCI: Tokyo Chemical Industry Co., Ltd. (Tokyo, Japan).

c Fujifilm Wako Pure Chemical Co. (Osaka, Japan);

d Sigma-Aldrich (MO, USA).

The HSPs of PLGA, PLA, PCL, and PSt were also determined using HSPiP based on the molecular group contribution method.^29^

### Calculation of RED between IR-1061 and Each Core Polymer

To evaluate whether IR-1061 has a high affinity for each core polymer, the distance between IR-1061 and the core polymer (*R*_a_) was calculated using eq 1.
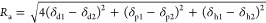
1Here, the subscripts 1 and 2 represent IR-1061 and each core polymer, respectively, and the δ values are the HSP factors discussed earlier. The RED provides an estimate of whether the two materials are miscible.^30^ RED is given by the ratio of *R*_0_ and *R*_a_, as shorn in eq 2.
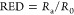
2A RED of 1 or less indicates that IR-1061 is expected to have a high affinity for the core polymer, whereas a RED larger than 1 means IR-1061 is expected to have a low affinity for the core polymer.

### Preparation of OTN-PNPs

The structure of the OTN-PNPs and the molecular structures of the components are shown in Figure 1. Briefly, PEG-*b*-PLGA in ACN (11.1 mg/mL, 9 mL) and IR-1061 in ACN (0.1 mg/mL, 1 mL) were mixed in a vial, followed by the addition to distilled water (40 mL). The mixing ratio of the polymer and the dye weights was set at 1000:1 based on the dependence of fluorescence intensity on the ratio investigated using PEG-*b*-PLGA (Figure S1). Then, the stirred aqueous solution was left overnight at 20 °C to allow the evaporation of the ACN. Finally, the obtained OTN-NIR fluorescent PEG-*b*-PLGA PNPs (OTN–PLGA-PNPs) were purified using a dialysis membrane (molecular weight cutoff (MWCO) = 2 kDa) overnight and then concentrated to 100 mg/mL using a centrifuge filter (MWCO = 100 kDa, 3000*g*, 20 min). OTN-NIR fluorescent PEG-*b*-PLA PNPs (OTN-PLA-PNPs) and PEG-*b*-PCL PNPs (OTN–PCL-PNPs) were prepared in the same manner, except that the block copolymers were changed.

### Characterization of OTN-PNPs

The sizes of the OTN-PNPs were determined using dynamic light scattering (LB-550, Horiba, Japan). The absorption spectra of the NP samples were measured using an ultraviolet–visible-NIR (UV–vis-NIR) spectrophotometer (V-770, JASCO, Japan). The fluorescence emission spectra of the OTN-PNP samples were measured under excitation with 980 nm light obtained from a xenon lamp set at 450 W in a spectrofluorometer (Fluorolog-3-21-NIR, Horiba, Japan) and a spectrometer (NIR-256-1.7; Avantes, Apeldoorn, Netherlands) equipped with a fiber-coupled diode (SP-976-5-1015-7; Laser Components GmbH, Olching, Germany) as the 980 nm excitation source.

### *In Vivo* OTN-NIR Fluorescence Imaging

Animal care and experiments were conducted according to the guidelines on the care and use of laboratory animals at the Tokyo University of Science under approval of the Tokyo University of Science’s Institutional Animal Care and Use Committee. Three week old female BALB/c mice were purchased from SLC Inc. (Shizuoka, Japan). Before the *in vivo* imaging experiments, the mice were fed the AIN-76A diet (Research Diets, New Brunswick, NJ, USA) for 2 weeks to reduce the levels of interfering phosphorescent compounds originating from alfalfa-based feed. Then, the mice were inoculated subcutaneously with colon-26 cells (1.0 × 10^5^ cells/mouse) in the side abdomen. Seven days later, the mice were anesthetized, and their hair was removed to prevent light scattering. Finally, 100 μL of OTN-PNP dispersion in PBS (50 mg/mL) was injected into the tail vein, and the OTN-NIR fluorescence images were captured using an OTN-NIR fluorescence *in vivo* imaging system (SAI-1000, Shimadzu, Japan).

## Results and Discussion

### Evaluation of the Affinity between IR-1061 and Core Polymers Using HSP Estimation

**Figure 2:**
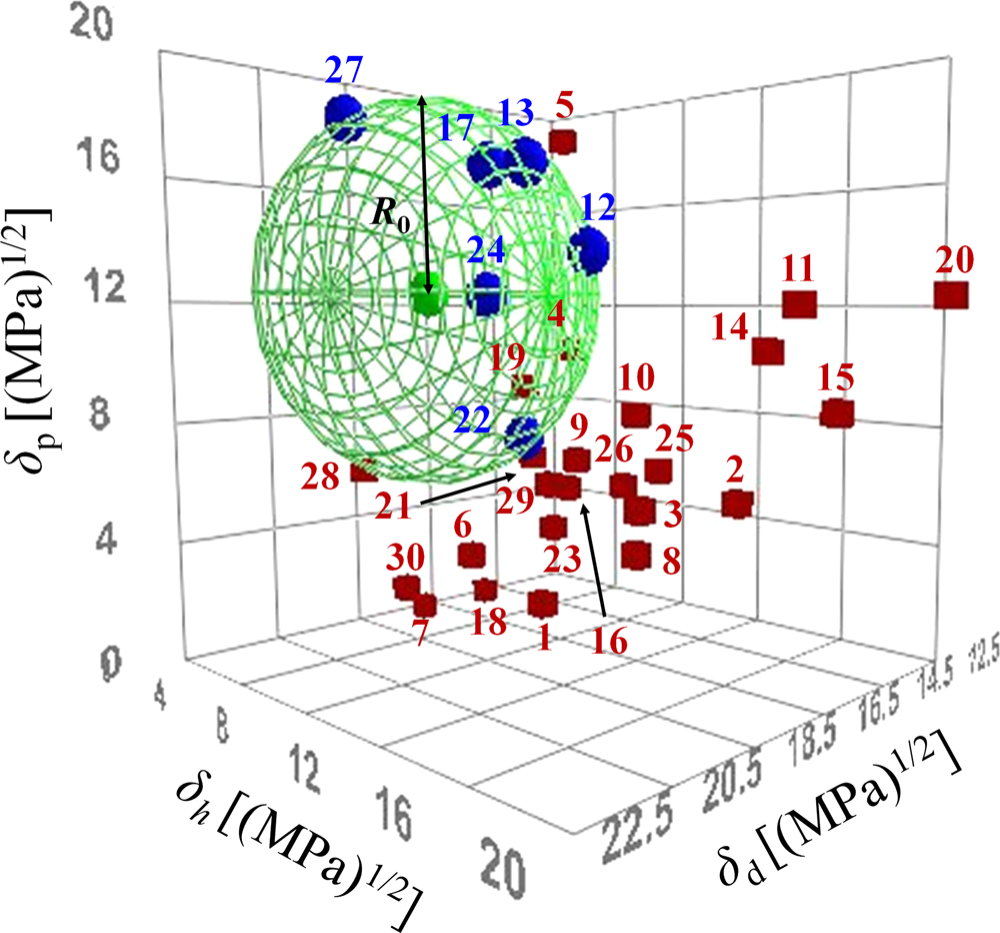
Three-dimensional plot of the HSP sphere of IR-1061 and the solubility parameters of organic solvents used in this work. Blue spheres and red cubes indicate the solvents that dissolved IR-1061 at a concentration of 2 mg/mL and those that did not, respectively. The red and blue numbers in the figure correspond to those in Table 1.

The HSP of IR-1061 was determined using the Hansen solubility sphere method. Table 1 shows the solubilities of IR-1061 in each organic solvent. *N*,*N*-Dimethylformamide, dimethyl sulfoxide, γ-butyrolactone, methylene dichloride, *N*-methyl-2-pyrrolidone, and propylene carbonate satisfied the criteria for dissolving IR-1061 at a concentration of 2 mg/mL. The Hansen solubility sphere plotted from the results of the solubility evaluation of IR-1061 is shown in Figure 2, in which the blue spheres and red cubes indicate the solvents that meet the solubility criteria (that is, those that dissolved IR-1061 at a concentration of 2 mg/mL) and those that do not, respectively. The HSP values of IR-1061 were established as follows: δ_d1_ = 18.8 [MPa]^1/2^, δ_p1_ = 12.3 [MPa]^1/2^, δ_h1_ = 5.7 [MPa]^1/2^, and *R*_0_ = 6.4 [MPa]^1/2^.

The HSPs of each core polymer were estimated by the molecular group contribution method. The RED values for IR-1061 with PLGA, PLA, PCL, and PSt are 0.56, 0.91, 1.55, and 1.91, respectively (Table 2). This means that PLGA and PLA, which have RED values of less than 1, can “dissolve” IR-1061; that is, they are highly compatible with IR-1061. In contrast, PCL and PSt have signifficantly different δ_p_ values from IR-1061. The obtained RED values indicate that the polarity of the core polymer is an important factor in designing or selecting a polymer that is highly compatible with IR-1061, which is consistent with our previous reports.^21^

**Table 2:** HSPs of the Core Polymers and RED between IR-1061 and Each Core Polymer

block copolymer	core polymer	δ_d2_ (MPa)^1/2^	δ_p2_ (MPa)^1/2^	δ_h2_ (MPa)^1/2^	*R*_a_ (MPa)^1/2^	RED
PEG-*b*-PLGA	PLGA	17.4	14.3	6.7	3.6	0.56
PEG-*b*-PLA	PLA	16.1	10.3	6.4	5.8	0.91
PEG-*b*-PCL	PCL	16.9	3.2	4.4	9.9	1.55
PEG-*b*-PSt	PSt	18.7	0.6	2.1	12.2	1.91

### *In Vitro* Properties of OTN-PNPs Containing PLGA, PLA, and PCL

**Figure 3:**
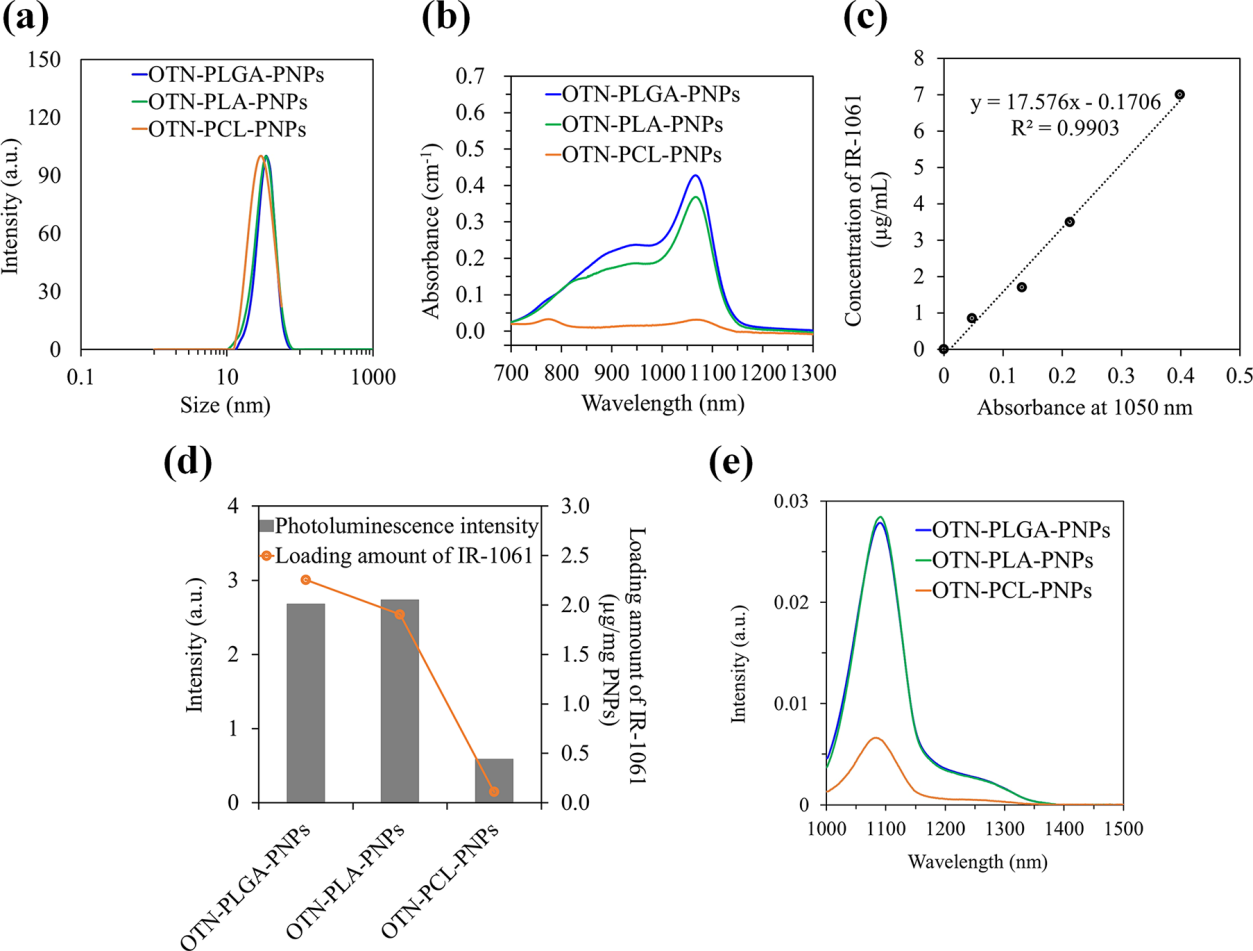
(a) Particle size distributions in PBS determined by dynamic light scattering measurements and (b) absorption spectra of OTN-PLGA PNPs, OTN-PLA PNPs, and OTN-PCL-PNPs (3 mg/mL in PBS). (c) Relationship between the concentration of IR-1061 in ACN and absorbance (cm^–1^) at 1050 nm. (d) Photoluminescence intensities of the OTN-PNPs (3 mg/mL in PBS) at 1100 nm and amount of IR-1061 loaded in the OTN-PNPs. (e) Emission spectra of the OTN-PNPs (3 mg/mL in PBS). Fluorescence measurements were carried out using laser excitation at 980 nm.

To validate the affinities predicted by HSP evaluation, we prepared three types of IR-1061-loaded polymer micelles having different core polymers, PLGA, PLA, and PCL. The OTN-PNPs were obtained by adding dropwise a mixture of IR-1061 and PEG-*b*-PLGA, PEG-*b*-PLA, or PEG-*b*-PCL in ACN to water, followed by stirring to remove the ACN and achieve micellization. The experiment using PSt, which showed the lowest result in the HSP prediction (Table 2), was not performed because we already reported that PSt alone has a low compatibility with IR-1061.^21^ The average particle sizes of the OTN-PLGA-PNPs, OTN-PLA-PNPs, and OTN-PCL-PNPs were 32, 32, and 39 nm, respectively (Figure 3a). The amounts of IR-1061 loaded in the OTN-PLGA-PNPs, OTN–PLA-PNPs, and OTN–PCL-PNPs were calculated from the absorption spectra (Figure 3b) and the concentration-dependent changes in the optical absorbance of IR-1061 (Figure 3c), and found to be 2.25, 1.90, and 0.11 μg/mg-PNPs, respectively (Figure 3d). The photoluminescence intensity of the OTN-PNPs was also different (Figure 3d, e) depending on the encapsulated amount of IR-1061. PLGA, PLA, and PCL are all insoluble in water, but their solubility parameters are very different. Particularly, PLGA and PLA have larger solubility parameters than PCL due to the difference in polarity and hydrogen bonding. This difference is expected to be related to the compatibility with IR-1061, which is similarly polar but insoluble in water. Thus, the amounts of IR-1061 encapsulated in the OTN-PLGA-PNPs and OTN-PLA-PNPs were significantly higher than that encapsulated in the OTN-PCL-PNPs, which is consistent with the differences in the affinities between IR-1061 and the core polymers based on HSP evaluation. It should be noted that the fluorescence intensity and absorption spectra used to calculate the amount of encapsulated dye are influenced by the surrounding molecules such as the core matrix. Therefore, the fluorescence intensity is not completely proportional to the dye amount calculated by the absorbance. Although the quantum yield of these PNPs was not evaluated, it was estimated from the NIR absorption data for OTN-PLGA-PNPs and OTN-PLA-PNPs to be similar to our previously reported polystyrene-based probes (approximately 0.6–0.7%).^21^

Interestingly, in the absorption spectrum of the OTN-PCL-PNPs, there is a peak at approximately 780 nm, suggesting that the IR-1061 dye was exposed to water (Figure 3b)^21^ and, thus, the PCL had not encapsulated IR-1061 well. In addition, the photoluminescence intensities of the OTN-PLGA-PNPs and OTN-PLA-PNPs are approximately 4 times higher than those of the OTN-PCL-PNPs, suggesting the usefulness of HSP prediction in designing highly emissive OTN-NIR fluorescent probes.

**Figure 4:**
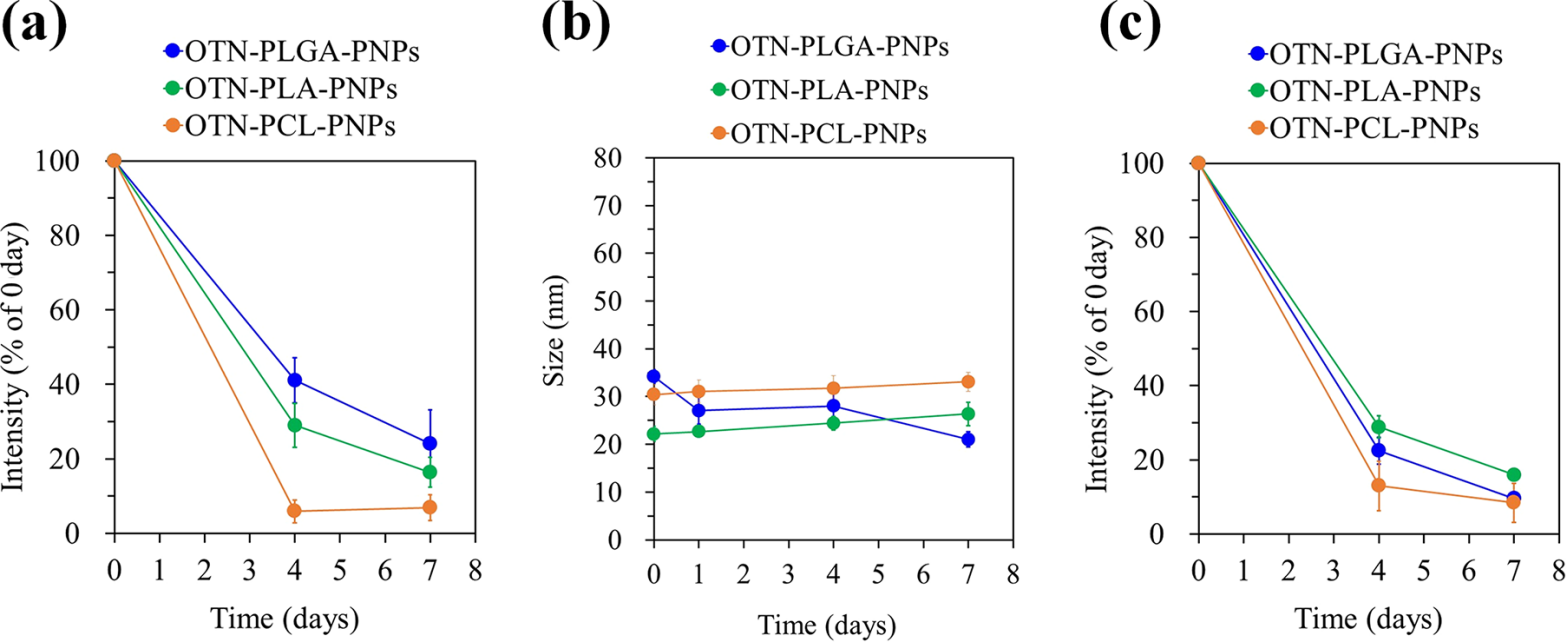
*In vitro* stabilities of OTN-PNPs with respect to (a) photoluminescence intensity in PBS at 37 °C, (b) particle size in PBS at 37 °C, and (c) photoluminescence intensity in 4% albumin in PBS at 37 °C. Data are shown as mean ± SD (*n* = 4/group).

The *in vitro* stabilities of the OTN-PLGA-PNPs, OTN-PLA-PNPs, and OTN-PCL-PNPs were assessed by analyzing the time-dependent changes in the OTN-NIR photoluminescence intensity of each OTN-PNP dispersed in PBS or a solution of 4% albumin dissolved in PBS at 37 °C for 7 days. The buffer and solution were used as *in vitro* models to test the stability of PNP *in vivo*, but it was tested for a longer period of time (up to 7 days) in the present study because these models were simpler than the actual *in vivo* environment and may have milder effects. The OTN-NIR photoluminescence intensities of the OTN-PLGA-PNPs, OTN-PLA-PNPs, and OTN-PCL-PNPs were maintained at 41 (±6.0)%, 29 (±5.9)%, and 5.9 (±3.1)%, respectively, after incubation in PBS for 4 days at 37 °C (Figure 4a). After a 7 day incubation, the retention rates were 24 (±9.0)%, 16 (±3.9)%, and 6.8 (±3.4)%, respectively (Figure 4a). This trend is consistent with the affinity of IR-1061 for the core polymers estimated from the RED values calculated from the HSPs. Because the PNPs showed high colloidal stability as the sizes of the particles remained almost constant during the test period (Figure 4b). The optical absorbance derived from IR-1061 decreased within 7 days in PNPs (Figure S2), suggesting that the decrease in photoluminescence is most likely a result of the leakage of IR-1061 from the core polymer. This leakage can be suppressed by matching the affinity between IR-1061 and the core polymer. On the other hand, in a solution of 4% albumin dissolved in PBS, the photoluminescence intensity decreased to approximately 25% after 4 days, even for the OTN-PLGA-PNPs and OTN-PLA-PNPs (Figure 4c). Albumin is the dominant protein in blood serum; thus, it was chosen as a model protein to estimate the influence of blood proteins on the stability of the OTN-PNPs. Free IR-1061 is completely quenched when it was just mixed with aqueous albumin solution owing to the influence of water molecules on IR-1061 (unpublished work). Because albumin interacts with the polymer micelles and, thus, reduces micelle stability,^31,32^ the albumin enhanced the leakage of IR-1061 from the core polymer. As above, the stability of OTN-PNPs was found to be different depending on the core polymer. Further studies are currently underway to analyze the kinetics of degradation and dye leakage by investigating their properties at more time points and to increase the stability of OTN-PNPs in the physiological environments.

### *In Vivo* Imaging Using OTN-NIR Fluorescence

**Figure 5:**
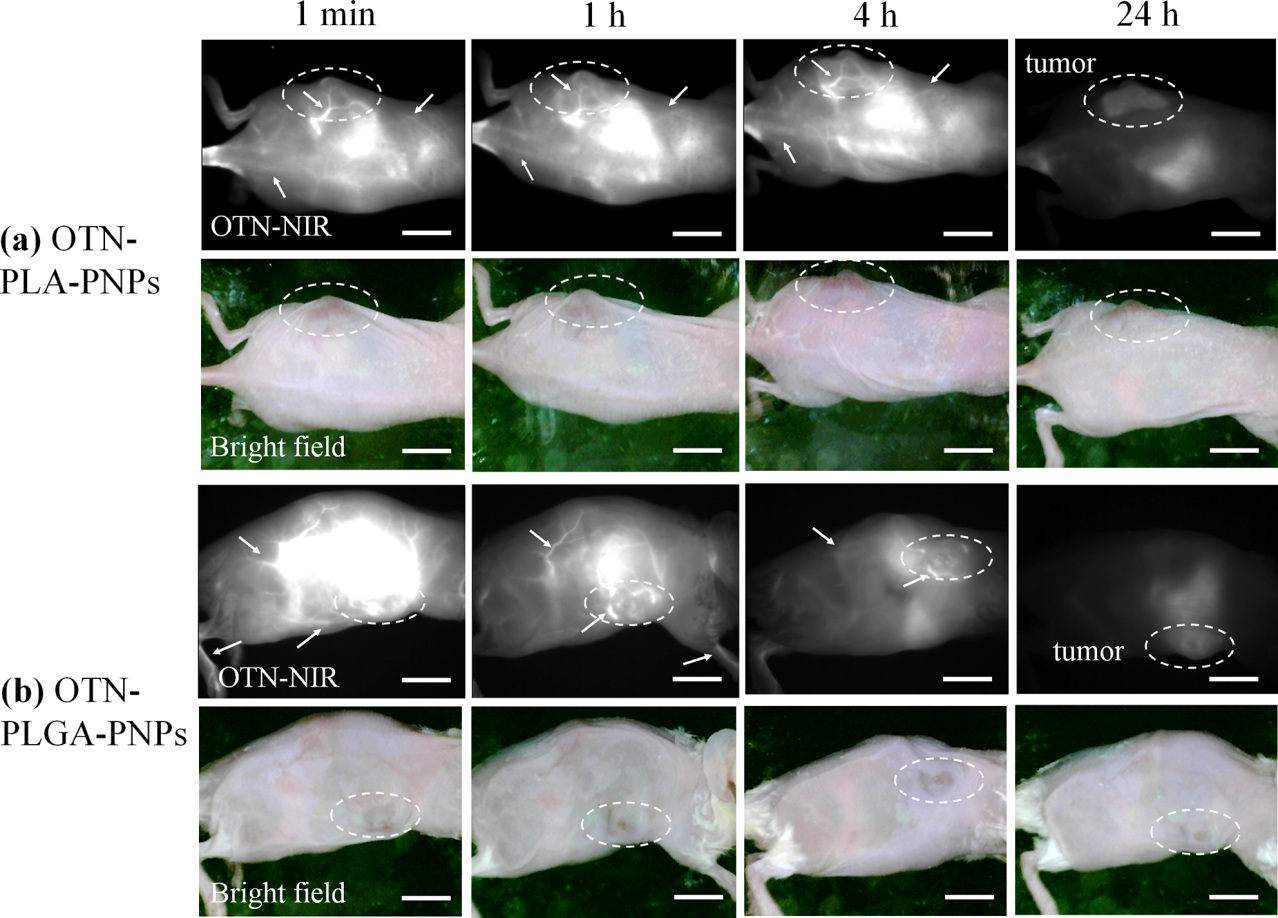
OTN-NIR fluorescence *in vivo* imaging of live mice. (a) OTN-PLA-PNPs (5 mg) and (b) OTN-PLGA-PNPs (5 mg) dispersed in PBS (0.1 mL) were injected intravenously into 6 week old female BALB/c mice inoculated with colon-26 cells. As a demonstration, the analysis was performed with two mice per group. Additional images are shown in Figure S3. The blood vessels (arrows) and the tumors (dotted circles) were visualized in OTN-NIR images observed under 980 nm light irradiation (0.4 W/cm^2^) with an integration time of 500 ms. Scale bars indicate 10 mm.

As a demonstration of the *in vivo* OTN-NIR fluorescence imaging capacity of the OTN-PLGA-PNPs and OTN-PLA-PNPs, we performed imaging of a cancer model lesion, which was established by subcutaneously inoculating colon-26 cells (a model cell line of murine colon carcinoma) into the abdomen. Immediately following the intravenous injection of the OTN-PNPs via the tail vein and excitation by light at 980 nm, the blood vessels under the skin could be observed with high clarity over the whole mouse body (Figure 5). Furthermore, imaging of the blood vessels was still possible 4 h after injection, indicating the high retention of the OTN-PLGA-PNPs and OTN-PLA-PNPs in the circulating blood. The OTN-NIR fluorescence emission was observed from the liver as a blood-rich organ as well as blood vessels until 4 h postinjection (Figure 5). Moreover, at 24 h after injection, the accumulation of OTN-PLGA-PNPs and OTN-PLA-PNPs in the tumors was observed (Figure 5), probably owing to the enhanced permeability and retention (EPR) effect.^33^ A decrease in image brightness over time after the injection of the OTN-PNPs was observed, but this is consistent with the results of the *in vitro* stability tests using albumin, and, as reported previously,^31,32^ OTN-PNPs typically decompose faster *in vivo* than in solutions containing a single protein because blood serum is a complex mixture of proteins, including albumins and globulins, as well as small molecular compounds.

In this study, the imaging analysis was performed with a single mouse per group as a demonstration to show the potential angiography and cancer imaging using our designed OTN-PNPs. For cancer imaging, the stability of encapsulated dye with PNPs should be controlled by design of the PNPs, because their long retention in the blood is generally required following intravenous injection. Thus, the polymer that encapsulates IR-1061 must not only be hydrophobic but also have a certain polar. This paper showed the OTN-PNPs with properties that enable cancer imaging can be designed by matching the compatibility of the dye with core polymers. On the basis of the *in vivo* study, the OTN-PNPs containing commercially available dyes and block copolymers designed based on their HSP can be used for both conventional vascular imaging^20,34^ and tumor imaging.

## Conclusion

We have reported a method to design OTN-NIR fluorescent-dye-loaded polymeric micellar nanoparticles (OTN-PNPs) by matching the solubility parameter of the core polymers to that of hydrophobic dye, which, in this study, was IR-1061. Using the Hansen solubility parameter, HSP, as an evaluation index, we found that PLGA and PLA have higher affinity for IR-1061 than PCL and PSt do. Further, the OTN-PNPs composed of PEG-*b*-PLGA and PEG-*b*-PLA showed higher IR-1061 encapsulation efficiencies, brightness, and stabilities in PBS compared to the OTN-PNPs composed of PEG-*b*-PCL, suggesting that, in the former cases, the dye molecules are etained in the PNP structure because of the higher affinity of IR-1061 for the core polymer. Crucially, using the OTN-PNPs composed of PEG-*b*-PLGA and PEG-*b*-PLA, the *in vivo* imaging of live mice could be performed, and blood vessels and tumor tissue could be imaged. Therefore, we proposed that matching the solubility parameters of dyes and core polymers is a useful approach for designing high-performance fluorescent polymer nanoparticles containing hydrophobic dyes.

## Supporting Information Available

Photoluminescence intensity of OTN-PNP; *in vitro* stability of OTN-PLGA-PNPs and OTN-PLA-PNPs; *in vivo* OTN-NIR fluorescence images (PDF)

## Abbreviations

ACN: acetonitrile
NIR: near-infrared
OTN: overthousand-nanometer
PBS: phosphate buffered saline
PCL: poly(ε-caprolactone)
PEG: polyethylene glycol
PLA: poly(lactic acid)
PLGA: poly(lactic-*co*-glycolic acid)
PNP: polymer micelle nanoparticles
PSt: polystyrene
