# Supplementary material for: Design of Over-1000 nm Near-Infrared Fluorescent Polymeric Micellar Nanoparticles by Matching the Solubility Parameter of the Core Polymer and Dye
Source: ACS Nanosci Au. 2021 Oct 4;1(1):61–8. doi: 10.1021/acsnanoscienceau.1c00010 (PMC10114859; doi:10.1021/acsnanoscienceau.1c00010)
Supplement: Supplementary file 1 — ng1c00010_si_001.pdf [file ng1c00010_si_001.pdf]

**Supporting Information**

# Design of Over-1000 nm Near-Infrared Fluorescent Polymeric Micellar Nanoparticles by Matching the Solubility Parameter of the Core Polymer and Dye

*Yuichi Ueya [1], Masakazu Umezawa [2,*], Yuka Kobayashi [1], Hisanori Kobayashi [2], Kotoe Ichihashi [2], Takashi Matsuda [1], Eiji Takamoto [1], Masao Kamimura [2], and Kohei Soga [2,*]*

1. Tsukuba Research Laboratories, JSR Corporation, 25 Miyukigaoka, Tsukuba, Ibaraki, 305-0841, Japan.
2. Department of Materials Science and Technology, Faculty of Advanced Engineering, Tokyo University of Science, 6-3-1 Niijuku, Katsushika, Tokyo 125-8585, Japan.

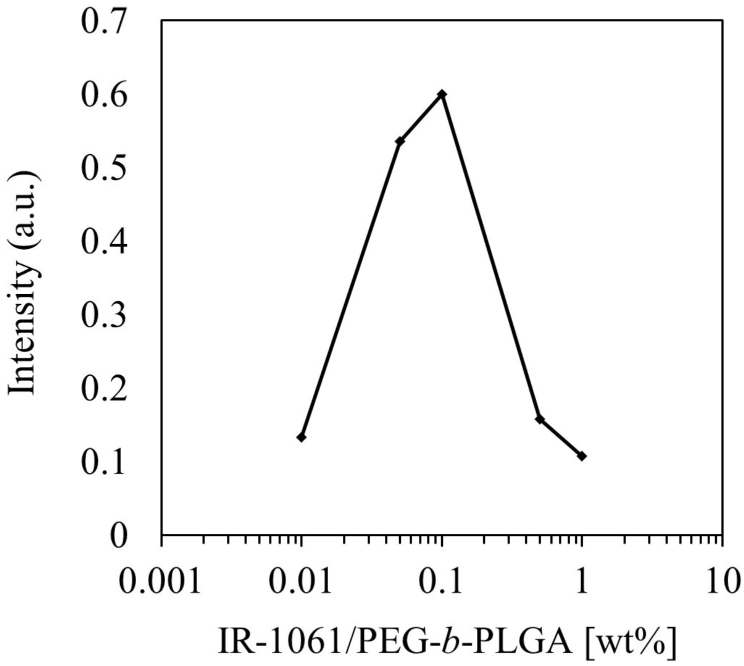


**Supplementary Fig. S1** – Photoluminescent intensity at 1100 nm (excitation: 980 nm) prepared with different mixture ratio of the dye (IR-1061) and the polymer (PEG-*b*-PLGA). The intensity of the OTN-PNP decreased via concentration quenching when it was prepared with too much dye and showed a peak when prepared with 1: 1000 (weight ratio) of IR-1061 and PEG-*b*-PLGA.

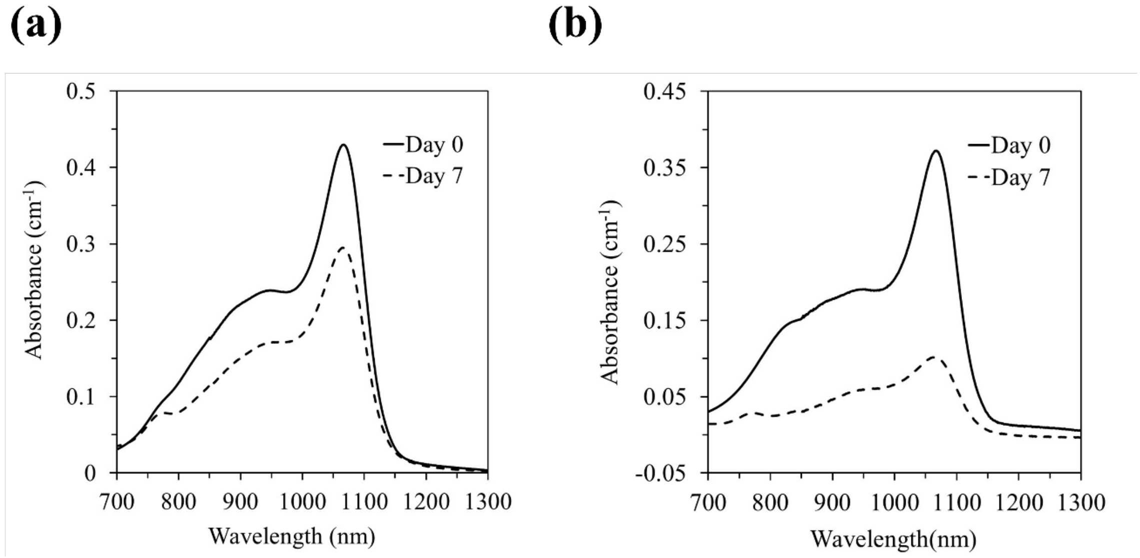


**Supplementary Fig. S2** – *In vitro* stability of (a) OTN-PLGA-PNPs and (b) OTN-PLA-PNPs (3 mg/mL in PBS) determined by the adsorption spectra at 37 °C for 7 days.

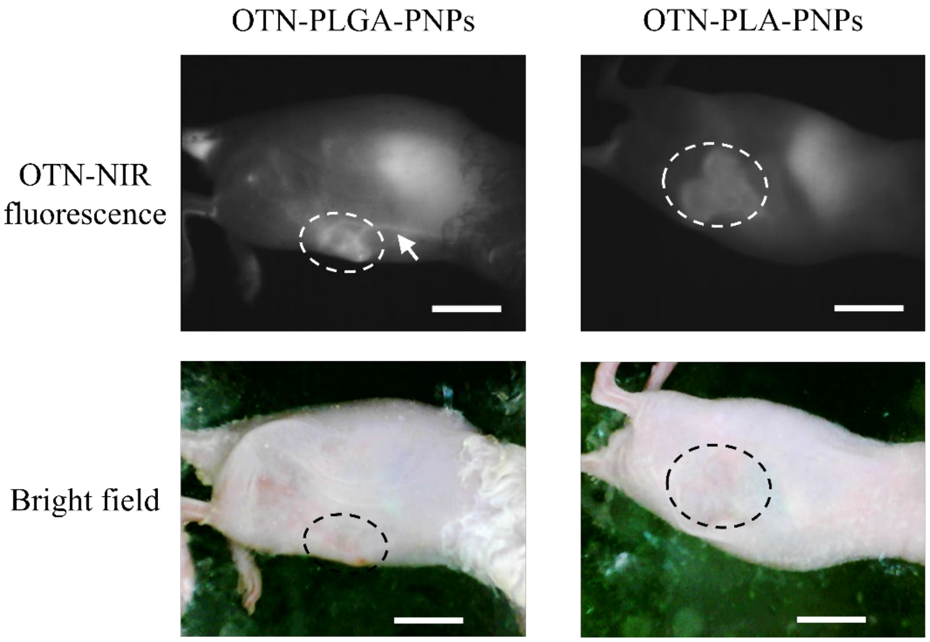


**Supplementary Fig. S3** – OTN-NIR fluorescence *in vivo* images of an additional mouse. (a) OTN-PLA-PNPs (5 mg) and (b) OTN-PLGA-PNPs (5 mg) dispersed in PBS (0.1 mL) were injected intravenously into six-week-old female BALB/c mice inoculated with colon-26 cells. At 4–24 h after the injection, the tumors (dotted circles) and blood vessel (arrow; ~4 h) were visualized in OTN-NIR images observed under 980-nm light irradiation (0.2 W/cm$^2$) with an integration time of 500 ms. Scale bars indicate 10 mm.
